# Automatic Spot Identification Method for High Throughput Surface Plasmon Resonance Imaging Analysis

**DOI:** 10.3390/bios8030085

**Published:** 2018-09-13

**Authors:** Zhiyou Wang, Xiaoqing Huang, Zhiqiang Cheng

**Affiliations:** 1 School of Electronic and Communication, Changsha University, Changsha 410003, China; moruyu3@gmail.com; 2 Department of Pharmacology and Molecular Sciences, Johns Hopkins University School of Medicine, Baltimore, MD 21205, USA

**Keywords:** surface plasmon resonance imaging, protein microarray, image enhancement, adaptive threshold binarization

## Abstract

An automatic spot identification method is developed for high throughput surface plasmon resonance imaging (SPRi) analysis. As a combination of video accessing, image enhancement, image processing and parallel processing techniques, the method can identify the spots in SPRi images of the microarray from SPRi video data. In demonstrations of the method, SPRi video data of different protein microarrays were processed by the method. Results show that our method can locate spots in the microarray accurately regardless of the microarray pattern, spot-background contrast, light nonuniformity and spotting defects, but also can provide address information of the spots.

## 1. Introduction

In the past few decades, the biomolecule microarray has emerged as a high-throughput parallel screening tool for the identification of biomolecular interaction and been widely applied in several fields, including functional protein screening [1,2], drug discovery [3,4], biomarker discovery [5] and antibody profiling [6,7]. Meanwhile, due to the obvious superiority of label-free, real-time monitoring and kinetic analysis, the surface plasmon resonance imaging (SPRi) biosensor has become an important technique for biomolecular interaction analysis (BIA) [8], and several methodologies have been carried out to enhance the sensitivities [9]. Up till now, SPRi has already combined organically with diverse biomolecule microarray contents and provided abundant dynamic information for the interaction association and dissociation, including DNA [10], protein [11,12,13,14], small molecule [15], peptide [16,17,18], peptoid nanosheet [19] and cell-based assays [20] in a high-throughput format.

In most SPRi instruments, an expanded and collimated light beam reflects from a metal slide supporting a microarray of biomolecules, and the reflection is collected as real-time images in video data by a charge-coupled device (CCD) detector array [21,22]. During the collection, the microarray is surveyed for interactions with a probe molecule in a high-throughput and parallel manner, and different reagents injected in the interactions can produce varied refractive index changes in spots of the microarray and their background [5]. In SPRi data analysis, the spots need to be identified accurately to measure the refractive index changes from the images. The greatest difficulty in the identification procedure is how to locate and address all the biomolecule spots in the microarrays from video data. Variations of the interaction strength and refractive index among the spots, as well as interruption factors, such as air bubbles, make it impossible to mark a report point of the image with the biggest spot-background contrast in the video. Thus, identifying the spots in the images randomly picked from video data cannot promise a desirable hit ratio. Besides, the following image quality issues could limit the accuracy of the spot identifications. On the one hand, light distribution through the SPRi images becomes nonuniform after the expansion and collimation. The nonuniformity of light deteriorates the image quality globally throughout the SPRi data analysis. On the other hand, spotting defects, such as spot missing, slide contamination, microarray misalignment and irregular contour of the spots, are introduced in the microarray fabrication procedures. The defects increase algorithm complexity and affect the accuracy of spot identification.

To achieve accurate spot identification, a number of methods have been reported [23]. To the best of our knowledge, most of the methods can be categorized in three main ways: (1) utilizing commercial microarray analysis software, such as Array Pro, GenePix Pro, MicroVigene, Dapple and, Mapix, along with the manufacturers’ microarray imaging instruments; the integration of instrument and analysis software brings convenience to users, but also strict constraints of data format and licensed usage [24]; (2) fitting shapes of the spots into circles or ellipses directly; many groups have developed types of algorithms based on the Hough transformation; in this transformation, pixels through the input image were required to be randomly picked in each ellipse fitting step, which was considered time consuming and susceptible to slide contamination [25]; and (3) detecting edges of the spots after image segmentation. The input image needs to be segmented into subgrids with only one spot inside each grid; thus, the accuracy of the type of methods can be affected by the spotting defects [26]. Moreover, the above methods have not been reported to identify the spots accurately from video data yet.

In this research, we proposed and developed a novel automatic spot identification method for high-throughput video format SPRi analysis. As a combination of video accessing, image enhancement, image processing and parallel processing approaches, our method can locate and address the protein spots in the SPRi video data by merging spots identified from all the images, regardless of the duration of measurements, reagent changes, light nonuniformity and spotting defects. To demonstrate the performance of the proposed method, we applied the method to SPRi video data of different microarray arrangements. In the test of a 3547 s-long SPRi video of a 12 × 12 microarray, it cost 84.47 s to identify all the spots when the maximal parallel processes are four. Meanwhile, standard deviations of the identified spot coordinates from all the images were less than one pixel. Our method shows sufficient accuracy of spot locations, but also can provide address information of the spots at a desirable running efficiency. As far as we know, our work is the first spot identification method for video data.

## 2. Materials and Methods

### 2.1. Materials

Protein A and phosphate-buffered saline (PBS) powder were purchased from Biosynthesis Biotechnology Co. and Solarbio Biological Co. respectively.FK506 binding protein (FKBP) 12 and rabbit IgG were purchased from Sino Biological Co., Ltd, Beijing, China. Thiol-poly(ethylene glycol) acid (HE003017-2K) was purchased from Hunan Huateng Pharmaceutical Co., Ltd, Changsha, China. N-(3-dimethylaminopropyl)-N-ethylcarbodiimide hydrochloride (EDC) and N-hydroxysulfosuccinimide sodium salt (sulfo-NHS) were purchased from Medpep. D263T optical glass (75 mm × 25 mm) was obtained from Daheng Optical Thin Film Co., Ltd, Beijing, China. FK506 was purchased from Sigma-Aldrich LLC, Shanghai, China. Chromium and gold for evaporation were obtained from the General Research Institute for Nonferrous Metals Co. Ltd, Beijing, China. All the other reagents were purchased from Beijing Chemical Industry Group Co. Ltd, Beijing, China.

### 2.2. Methods

Fabrication of gold slides, preparation of FKBP12 and rabbit IgG microarrays and SPRi measurements were implemented according to our previous works [27,28]. Briefly, the gold slides were modified by thiol-poly(ethylene glycol acid) by incubation in 4 ${}^{\circ }$C overnight and then activated by the sulfo-NHS/EDC mixture (0.1 M and 0.2 M, respectively). After that, 1 mg/mL of proteins were spotted onto the slides by a Sci Flexearryer DW (Scienion Ag Co., Berlin, Germany) with a designed pattern. The FKBP12 microarray contains 12 × 12 800 μm-diameter spots, and rabbit IgG microarray contains 36 × 36 200 μm-diameter spots. The slides were finally incubated in 5 mg/mL milk PBS solution to block the unprinted area and reduce the non-specific bindings. The slides were mounted on an SPRi KX5 system (Plexera, LLC) and monitored by a CCD with a 10 μm × 10 μm area in each pixel. Images in SPRi video data were captured at a speed of 1 frame/second. In the SPRi measurements of FKBP12 and rabbit IgG microarrays, FK506 and protein A were utilized as the probe molecules, respectively. The reported spot identification method was developed in Python Version 3.5 for Windows ×64 on a computer with an Intel Core i3 3.70-GHz CPU and 4 GB RAM running Windows 7 Ultimate operating system.

**Figure 1:**
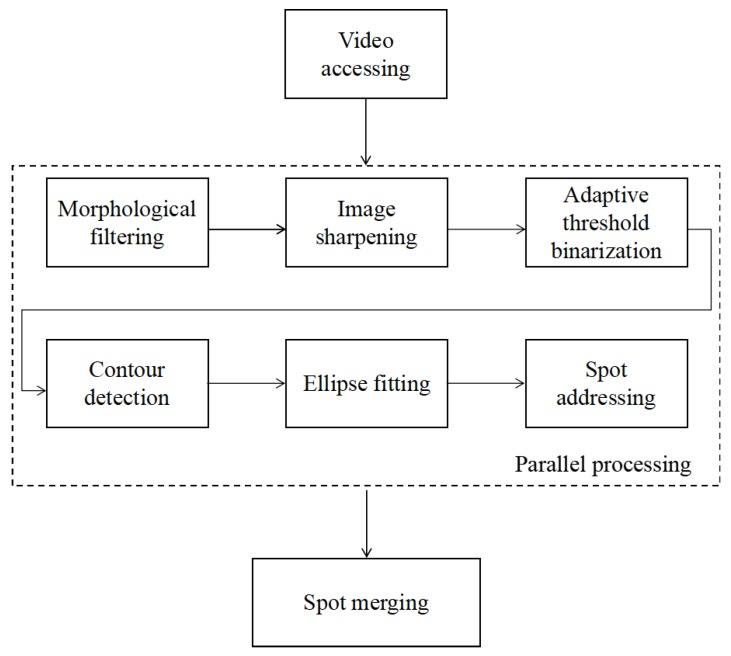
Flowchart of the automatic spot identification method.

The proposed spot identification method is composed of four parts: video accessing for image reading from the SPRi video data, image enhancement for modifying attributes of the captured images and image processing for spot identification. The image enhancement part includes morphological filtering, contrast normalization and image sharpening methods, while the image processing part includes adaptive threshold binarization, contour detection and ellipse fitting methods. Identified spots from all the images are merged into a set of spots for the SPRi video data in the spot merging step. To promote the running efficiency of the image enhancement and processing algorithms, images from the video were assigned to parallel processes of the CPU. After being located by the ellipse fitting methods, all the spots located from the images were addressed and merged by coordinates into a new spot set as identification results for the video data. Figure 1 shows a flowchart illustrating the steps of the spot identification method, and the steps are described separately in the following subsections.

#### 2.2.1. Video Accessing and Parallel Processing

To allow all the images in SPRi video data to be processed in a parallel way, the video handle of the data was obtained by the VideoCapture function in the Open Source Computer Vision Library (OpenCV) [29,30,31]. Afterward, all images in the video data were read from the handle and processed by the following image enhancement and processing algorithms assigned to different asynchronous processes by using multiple processing packages of Python. Sets of identified spots from each image were returned by the corresponding process and stored in memory for the spot addressing and merging step.

#### 2.2.2. Morphological Filtering

**Figure 2:**
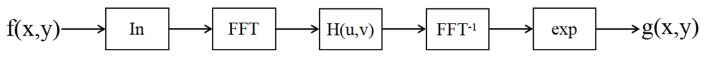
Flowchart of homomorphic filtering.

To overcome the light nonuniformity limit in the images, homomorphic filtering and image erosion algorithms of the morphological filtering family were utilized separately [32,33]. The flowchart of the homomorphic filtering is shown in Figure 2.

Firstly, we took the natural logarithm of the images captured by the SPRi platform (*f(x,y)* in Figure 2) and applied fast Fourier transformation (FFT) to the logarithm data. Then, we designed the spatial domain homeostatic filter *h(x,y)* and made FFT obtain the frequency domain homeostatic filter *H(u,v)*. We transformed the filtered data to the spatial domain and calculated their exponential values to get homomorphic filtered images, where the width and height parameters are the width and height of the SPRi images, respectively, and σ is the standard deviation parameter determining the size of the filter kernel.

(1) $$h\left(x,y\right)=1-\exp\left(-\frac{{\left(x-width/2\right)}^{2}+{\left(y-height\right)}^{2}}{2\sigma^{2}}\right)$$

After the homomorphic filtering, the contamination spots introduced by spotting can be suppressed by the image erosion algorithm. The erosion operator takes two inputs, an image to be eroded and a small-sized array as the erosion kernel. In this step, we designed a flat disk of suitable size for grayscale erosion, which can erode small bright noise areas in the dark background of the images down to their surrounding intensity value [29]. Spots with can irregular contour can be compensated as ellipses in the images by the image dilation algorithm. Similar to the erosion algorithm, the dilation operator also takes two inputs, the image to be dilated and a small-sized array as the dilation kernel. In this step, we designed a flat disk of a suitable size for dilation, which can fill in irregular contours of the spots, to be the surrounding intensity value [29].

#### 2.2.3. Image Sharpening

After the morphological filtering step, edges of the spots in the images need to be sharpened to reduce the complexity of the later image processing algorithms. Based on the principle of OpenCV function filter2D, we designed a 2D array with an odd number of rows and columns as the Laplacian convolution kernel. In the array, the central element corresponds to the weight of the pixel of interest, and the other elements correspond to weights of the pixel’s neighbors. When contrasting between the weight of the pixel and its neighbors increases, edges of the spots in the images filtered are sharper [29,34,35].

#### 2.2.4. Adaptive Threshold Binarization

To separate pixel values into two groups, white as spot areas and black as background areas, the enhanced images need to be binarized. In the binarization process, thresholding plays a major role [36,37]. Considering the images of different global threshold values need to be processed automatically, we turn to the adaptive thresholding strategy in the OpenCV library instead in the binarization process. In this strategy, different regions of block sizes in the different images can be calculated by the algorithm, resulting in a desirable binarization effect.

#### 2.2.5. Contour Detection, Ellipse Fitting and Spot Addressing

Contour, which is a curve joining all the continuous points along the boundary of the same color or intensity, is a useful tool for the spot detection and recognition [38,39,40,41]. In the findcontours function of the OpenCV library, we tried different finding modes and found that the “RETR-CCOMP” mode is the best choice. In this mode, only the contours of the highest hierarchy in the images are found for the later ellipse fitting step. The Fitellipse function in the OpenCV library was utilized to fit the found contours of the spots into ellipses. The coordinate of the ellipse’s center pixel is the location of the spot, while horizontal and vertical radii of the ellipse are the width and height of the spot, respectively. Each set of the spots from the image were categorized into different rows and columns by their coordinates, and adjacent blank areas were selected as their background.

#### 2.2.6. Spot Merging

The mean value and standard deviations of spots at the same column and row address among all the images were calculated. If the standard deviation at one column and row address was smaller than 1 pixel, then spots at the address in all the images were considered at the same location. In this case, the spot in the first image of the SPRi video was added to the set of the identified spots. If the standard deviation at one column and row address was bigger than 1 pixel, the spot with the biggest deviation from the mean value was added to the set of identified spots. The set of spots in this step was addressed as the final results of spot identification in the SPRi video data.

## 3. Results and Discussion

### 3.1. FKBP12 Microarray

**Figure 3:**
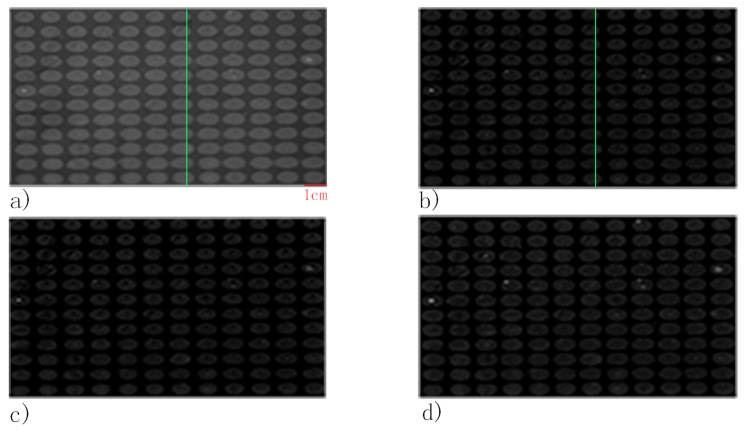
Image of the FKBP12 microarray (**a**) image from SPRi; (**b**) after homomorphic filtering; (**c**) after erosion and (**d**) after dilation.

**Figure 4:**
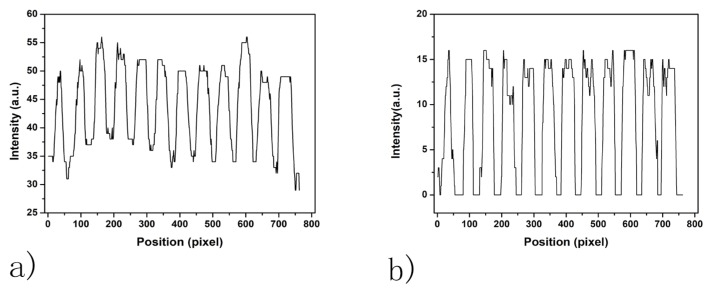
Line section in the SPRi image of the FKBP12 microarray (**a**) before homomorphic filtering and (**b**) after homomorphic filtering.

**Figure 5:**
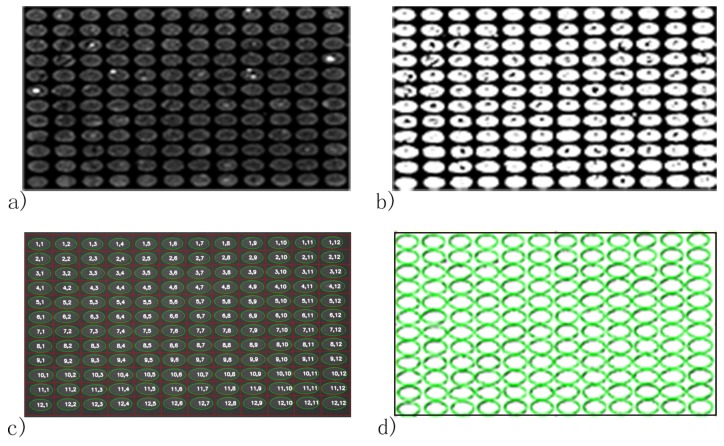
Spot identification results of the FKBP12 microarray by the automatic spot identification method and Ellipse Split plugin of ImageJ. (**a**) Image of the FKBP12 microarray after the sharpening procedure; (**b**) adaptive threshold binarization of the image; (**c**) spot location and addressing result of the automatic spot identification method and (**d**) spot location result of the Ellipse Split plugin of ImageJ.

The average spot-background contrast was 1.29 in the SPRi image at PBS injection as shown in Figure 3a. Due to the image being compact in the vertical direction of the SPRi video data, all the spots in the microarray were ellipses. The length of the major axis of each spot was slightly larger than 800 μm, which was caused by diffusion of protein in the incubation step. The line section of Figure 3a is shown in Figure 4a. The curvature of the baseline in Figure 4a indicates that light was nonuniform through the image [28]. Besides, irregular and unclear contours of the spots and slide contamination can be seen in the figure. After the homomorphic filtering, the image and its line section were as shown in Figure 3b and Figure 4b, respectively. The straight baseline in Figure 4b indicates that the nonuniformity of light was eliminated by homomorphic filtering. In Figure 3c, slide contamination was suppressed by the image erosion algorithm. After the image dilation and sharpening, all the spots presented clear ellipse contours suitable for the later image processing algorithms in Figure 5a. Figure 5b shows the binarized image produced by the adaptive threshold binarization algorithm. In Figure 5c, locations, outlines, addresses and background of all the spots can be identified by the ellipse fitting perfectly.

As a comparison, the Ellipse Split plugin (Version 0.5.0, http://imagej.net/Ellipse_split) of ImageJ (Version 1.52a) was utilized to identify the spots in Figure 3a [42]. Results in Figure 5d indicate that locations of the spots identified by our method agree well with the plugin of ImageJ. However, the plugin failed to calculate the addresses and background of the spots. In a further comparison of calculation speed, 100 images of the microarray were processed by the two methods; the calculation time of the Ellipse Split plugin of ImageJ was 68.687 s, while that of the proposed method was 2.466 s, when the number of processes was four. The above comparisons show that our method can be 27.85-times faster than the Ellipse Split plugin of ImageJ.

**Figure 6:**
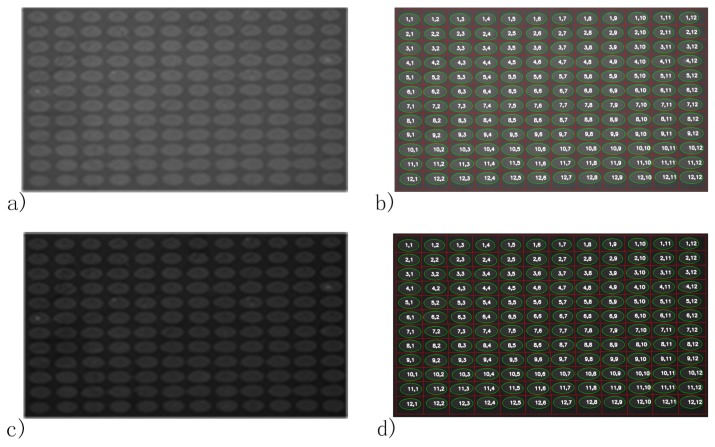
Automatic spot identification results of the FKBP12 microarray for different reagent injections. (**a**) SPRi image of the FKBP12 microarray for the FK506 injection; (**b**) automatic spot identification result of the microarray for the FK506 injection; (**c**) SPRi image of the FKBP12 microarray for the phosphorous acid injection and (**d**) automatic spot identification result of the microarray for the phosphorous acid injection.

**Figure 7:**
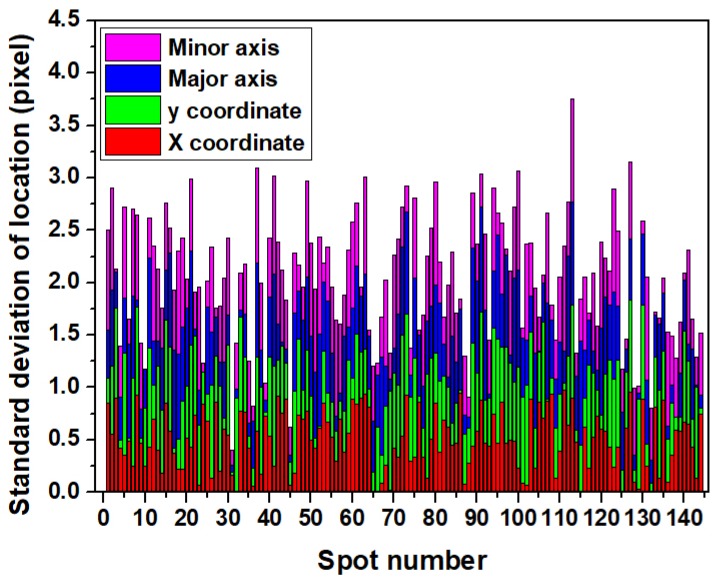
Standard deviation of the x coordinate, y coordinate, major axis and minor axis of spots identified in all images from SPRi video data of the FKBP12 microarray.

To further test the effect of reagent changes on our spot identification method, the reported method was applied to images of FK506 and phosphorous acid injections, as in Figure 6. As shown, though the average intensity of background areas in Figure 6a (spot-background contrast: 1.22) and Figure 6c (spot-background contrast: 1.50) were different from Figure 3a, the locations, addresses and background of all the spots can be identified as in Figure 6b,d. Spot location deviations of FK506 and phosphorous acid injections via PBS injection in Table 1 demonstrate that our automatic spot identification method worked well regardless of spot-background for different reagents during SPRi measurements. With there being four processes, the proposed method was applied to the SPRi video of the microarray (3547 s long), and it costs 84.47 s to identify the spots from all the images. Standard deviations of coordinates, the major axis and minor axis of all the spots were estimated as in Figure 7. All the standard deviations of all the spots were smaller than one pixel; thus, the spots in Figure 5c are the set of spots identified from the SPRi video data of the FKBP12 microarray.

### 3.2. Rabbit IgG Microarray

**Figure 8:**
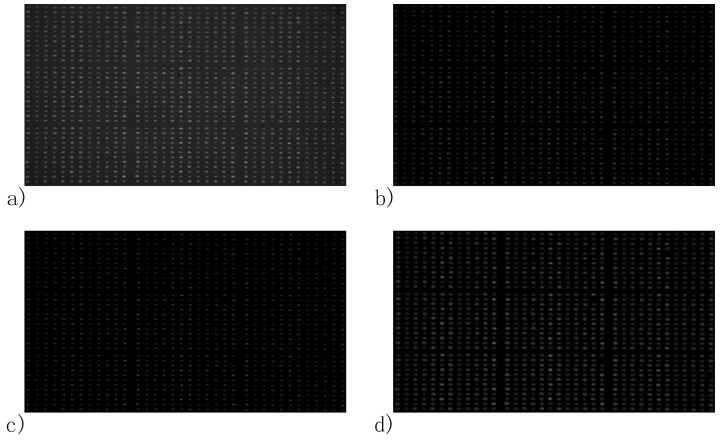
Image of the rabbit IgG microarray (**a**) from SPRi measurement; (**b**) after homomorphic filtering; (**c**) after image erosion and (**d**) after image dilation.

**Figure 9:**
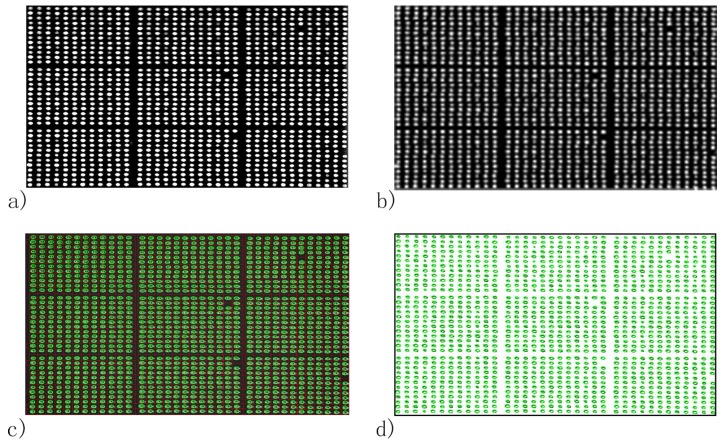
Spot identification results of the rabbit IgG microarray by the automatic spot identification method and Ellipse Split plugin of ImageJ. (**a**) Image of the rabbit IgG microarray after the sharpening procedure; (**b**) adaptive threshold binarization of the image; (**c**) spot location and addressing result of the automatic spot identification method and (**d**) spot location result of the Ellipse Split plugin of ImageJ.

To test the spot identification applicability in high-throughput microarray analysis, the reported method and the Ellipse Split plugin of ImageJ were applied to the image of the rabbit IgG microarray of PBS injection, respectively. In the following work, we define blocki,j as the block in row i and column j and spotl,m as the spot in row l and column m in a block. In Figure 8a, spot 5,7 in block 1,3, spot 2,11 in block 2,2, spot 2,12 in block 3,2 and spot 4,12 in block 3,3 were missed in the spotting step. Besides, misalignment of the microarray introduced by the spotting step, slide contamination in the background and nonuniformity of light can be seen in the figure. After being processed by the above image enhancement algorithms of our method, except the spots missed in the spotting step, all the spots in the microarray presented as clear and regular ellipses in Figure 9a. The result of the ellipse fitting algorithm in Figure 9c shows that outlines, locations, addresses and the background of the spots identified agree well with the results of the Ellipse Split plugin in Figure 9d. The above spot identification results of FKBP12 and rabbit IgG microarrays show that the proposed automatic spot identification method worked well for SPRi video data regardless of microarray pattern, slide contamination, spot missing, nonuniformity of light in images, irregular contours of spots and misalignment of microarrays.

## 4. Conclusions

This work proposed and developed an automatic spot identification method for the SPRi biosensor. As a combination of video accessing, image enhancement, image processing and parallel processing techniques, the method can locate and address spots from video data accurately regardless of the microarray pattern, slide contamination, spot missing, nonuniformity of light, irregular contours of spots, spot-background contrast and misalignment of microarrays with a desirable running efficiency. To make our method a more advanced tool for high throughput protein microarray analysis of the SPRi platform, the method will be integrated with intensity and dynamic analysis techniques in the future.

## Figures and Tables

**Table 1:** Average deviations of spot locations for FK506 and phosphorous acid injections via PBS injection in the automatic spot identification method.

	X Coordinate (Pixel)	Y Coordinate (Pixel)	Major Axis (Pixel)	Minor Axis (Pixel)
FK506	−0.195	−0.193	−0.164	0.243
Phosphorous Acid	−0.241	0.475	0.304	−0.710
